# Identification and characterization of the novel reversible and selective cathepsin X inhibitors

**DOI:** 10.1038/s41598-017-11935-1

**Published:** 2017-09-13

**Authors:** Urša Pečar Fonović, Ana Mitrović, Damijan Knez, Tanja Jakoš, Anja Pišlar, Boris Brus, Bojan Doljak, Jure Stojan, Simon Žakelj, Jurij Trontelj, Stanislav Gobec, Janko Kos

**Affiliations:** 1 Faculty of Pharmacy, University of Ljubljana, Aškerčeva 7, Ljubljana, Slovenia; 2 Institute of Biochemistry, Faculty of Medicine, University of Ljubljana, Vrazov trg 2, Ljubljana, Slovenia; 3 Department of Biotechnology, Jožef Stefan Institute, Jamova 39, Ljubljana, Slovenia

## Abstract

Cathepsin X is a cysteine peptidase involved in the progression of cancer and neurodegenerative diseases. Targeting this enzyme with selective inhibitors opens a new possibility for intervention in several therapeutic areas. In this study triazole-based reversible and selective inhibitors of cathepsin X have been identified. Their selectivity and binding is enhanced when the 2,3-dihydrobenzo[*b*][1,4]dioxine moiety is present as the R^1^ substituent. Of a series of selected triazole-benzodioxine derivatives, compound **22** is the most potent inhibitor of cathepsin X carboxypeptidase activity (K_i_ = 2.45 ± 0.05 μM) with at least 100-fold greater selectivity in comparison to cathepsin B or other related cysteine peptidases. Compound **22** is not cytotoxic to prostate cancer cells PC-3 or pheochromocytoma PC-12 cells at concentrations up to 10 μM. It significantly inhibits the migration of tumor cells and increases the outgrowth of neurites, both processes being under the control of cathepsin X carboxypeptidase activity. Compound **22** and other characterized triazole-based inhibitors thus possess a great potential for further development resulting in several *in vivo* applications.

## Introduction

Cathepsin X is a carboxypeptidase expressed predominantly in immune and neuronal cells^1^. It regulates their migration, adhesion, proliferation, and maturation, together with phagocytosis and signal transduction^2^. Over the last ten years several molecules have been identified as substrates for its carboxypeptidase activity, including the β-chain of integrin receptors, γ-enolase, chemokine CXCL-12, bradykinin, kallidin, huntingtin and profilin 1^3^. Besides its enzymatic activity the other mode of action is via the RGD motif that is part of the prodomain that binds to integrin receptors and extracellular matrix proteins^1, 3^. Since increased expression of cathepsin X and/ or activity is associated with certain types of cancer^4–8^, neurodegenerative disorders^9^, inflammatory^10^ and other diseases^3^, it is considered to be a promising target for the development of new therapeutic agents.

The number of therapeutically useful inhibitors for cathepsin X is, however, very limited. The well-known irreversible inhibitor of cysteine cathepsins E-64, isolated from *Aspergillus japonicus* ^11^, is a very weak inhibitor of cathepsin X (k_inact_/K_i_ = 775 M^–1^s^–1^)^12^. *n*PrNH-(*2 S*,*3 S*)tEps-Ile-OH, designed to place a single amino acid residue in the S’ subsite of cathepsin X, has a 10-fold preference over cathepsins B or L, however, it still exhibits a relatively weak inhibitory potency toward cathepsin X (k_inact_/K_i_ = 225 M^–1^s^–1^)^13^. Another epoxysuccinyl-based inhibitor of cathepsin X is AMS36 which has a naphthalene methylamine in P3 and a non-natural *p*-methyl phenylalanine moiety in the P2 position^14^. In tumor tissues, AMS36 shows selectivity for cathepsin X but, in other tissues such as rat liver and kidney, a significant cross-reactivity with cathepsin B was observed^14^. Nevertheless, AMS36 is frequently used as a cathepsin X specific inhibitor, usually in parallel with the cathepsin X specific 2F12 monoclonal antibody^15^ or cathepsin X specific siRNA silencing^16, 17^.

Here, triazole-based reversible cathepsin X inhibitors, resulting from an in-house compound library screening, are described. They exhibit values of K_i_ in the low micromolar range, high selectivity for cathepsin X, and are not cytotoxic for PC-3 prostate cancer cells or PC-12 pheochromocytoma cells. The inhibitors were further evaluated in cell based assays where their impact on tumor cell migration and neurite outgrowth, two processes strongly associated with increased cathepsin X activity, was established.

## Results

### Relative inhibition of cathepsin X

**Figure 1:**
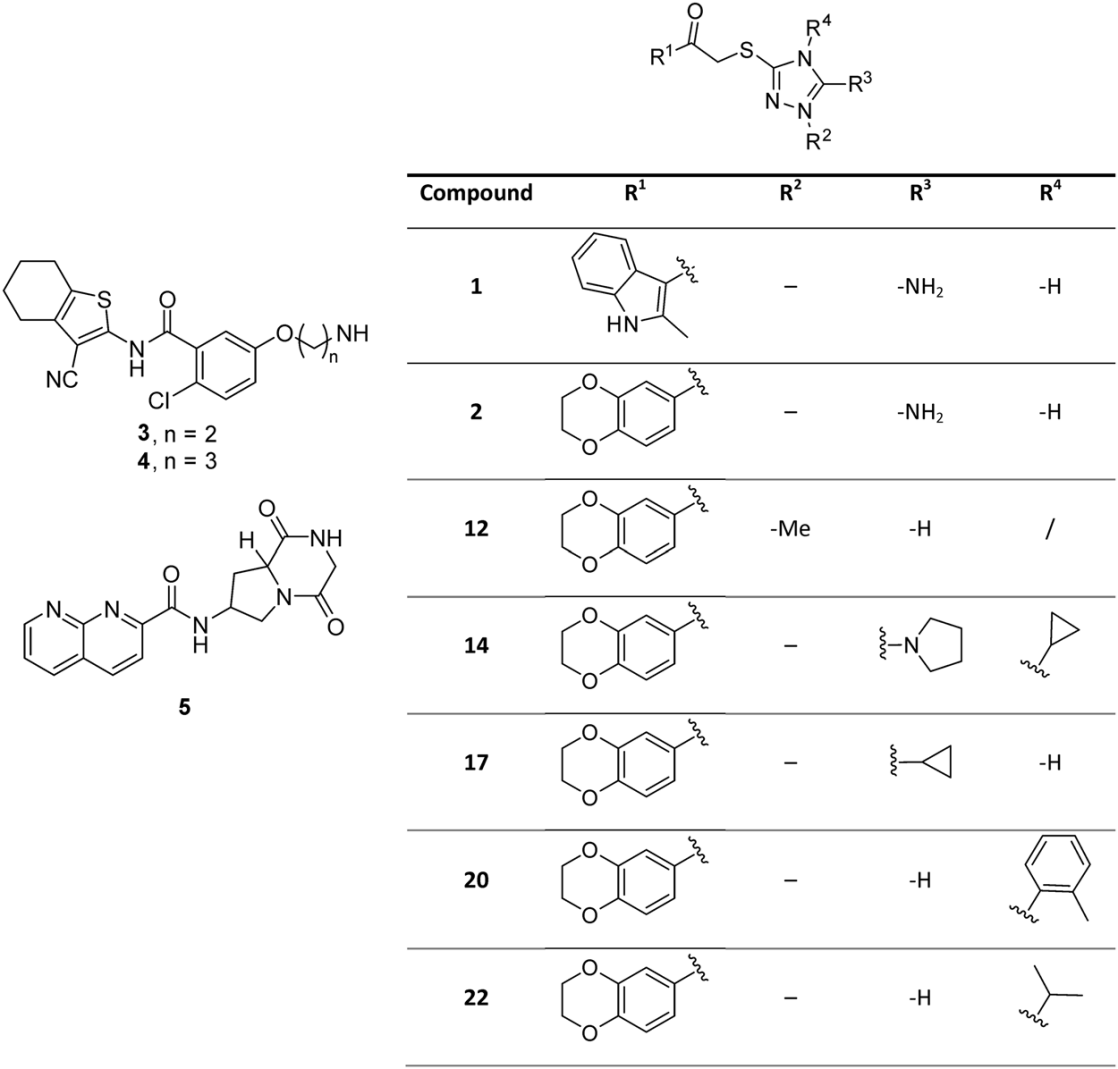
Structures of the selected cathepsin X inhibitors.

579 diverse and drug-like compounds^18^ in a chemical library obtained from different vendors or synthesized in-house (the first group) were tested for their inhibition of cathepsin X. From the initial screening at 50 μM concentration, several compounds were excluded due to spectral interferences at the wavelength used to follow the enzyme reaction. Compounds exhibiting less than 50% inhibition were also excluded. The five remaining compounds **1**, **2**, **3**, **4** and **5** exhibited a relative inhibition between 60 and 75% (Fig. 1, Supplementary Fig. S1). After determining the value of K_i_ (see below) two hits from the first group, both having a triazole ring in their structure (compounds **1** and **2**), were selected for further evaluation. Based on structural similarity, a new group of twenty “analogues by catalogue” compounds (the second group) having a triazole scaffold with variations at the R^1^ position and various substituents on the triazole ring (Supplementary Table S1, Fig. 1) was purchased from commercial sources (Enamine Ltd. and ChemBridge Corp.). Again, relative inhibition was determined, and a group of 5 compounds (**12**, **14**, **17**, **20** and **22**) inhibiting cathepsin X by more than 70% was selected for further studies (Supplementary Fig. S1). All had a 2,3-dihydrobenzo[*b*][1,4]dioxine fragment as R^1^ substituent.

### Selected inhibitors bind reversibly to cathepsin X

**Figure 2:**
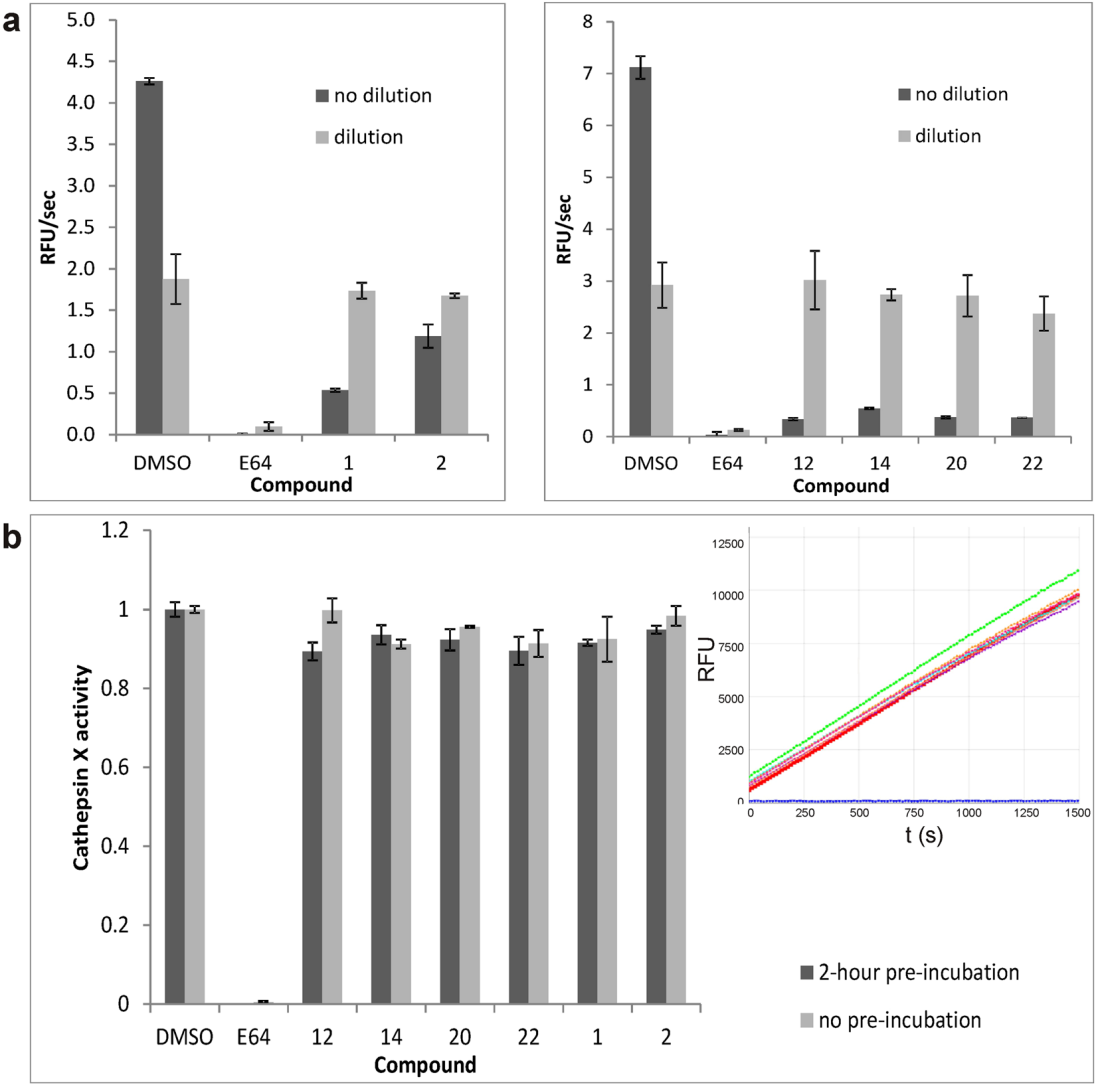
Selected inhibitors bind reversibly to cathepsin X. (**a**) Cathepsin X activity was measured immediately (dark grey) after 1-hour pre-incubation with selected compounds, or after 200000-fold dilution in activation buffer using a Centricon (10 kDa cut-off; Millipore) for washing out the compounds (light grey). After removing the compounds almost all cathepsin X activity was regained according to the control (DMSO) except in the case of irreversible inhibitor E-64. Values are means of two independent experiments, performed in duplicate ± S.E.M. (**b**) After rapid 100-fold dilution of (pre-incubated) cathepsin X (100-fold concentration) and compound (10-fold concentration) cathepsin X activity was measured. 90% of the recovered cathepsin X activity points toward reversible inhibitors with rapid dissociation. Inset: graph representation of activity measurements - control (green), E-64 (blue) and tested compounds (other colors).

The reversibility of inhibition of cathepsin X was determined by the washout experiment and the dilution method. After 1-hour pre-incubation with the enzyme, tested compounds inhibited cathepsin X to a similar extent as in the initial experiments without the pre-incubation. After a 200000-fold dilution and removal of the inhibitors through a membrane with a cut-off of 10 kDa, the cathepsin X activity was almost completely recovered and similar to that of the DMSO control (Fig. 2a). During the 4 hour experiment, the cathepsin X activity of control samples and of samples incubated with tested inhibitors fell by 50%. The irreversible inhibitor E-64 was used as a positive control and retained cathepsin X inhibition after the washout. Similar results were obtained after a 100-fold rapid dilution of the reaction mixture, when approximately 90% of the enzyme activity was recovered (Fig. 2b), confirming that the tested compounds are reversible inhibitors with rapid dissociation^19^. A control sample containing E-64 remained inhibited, as expected for an irreversible inhibitor.

### K_i_ values and specificity of compounds

Inhibition constants (K_i_ values) for the hit compounds from both groups were determined at three different substrate concentrations (Supplementary Table S2). In the first group, K_i_ values and the mechanism of inhibition were analyzed by non-linear regression using SigmaPlot software. Inhibitors displayed K_i_ values in the low micromolar range (Table 1). The most potent inhibitors of cathepsin X were compounds **1** and **2** (K_i_ = 38.4 ± 0.20 μM and 29.2 ± 2.44 μM, respectively).

**Table 1:** K_i_ values against cathepsin X and the mechanism of inhibition.

Compound	Ki (μM)	
1*	38.40 ± 0.20	
2*	29.20 ± 2.44	
3*	259.74 ± 31.87	
4*	230.21 ± 2.90	
5*	135.90	
**Compound**	**Ki (μM)**	**Inhibition mechanism**
12	5.88 ± 0.21	competitive
14	5.41 ± 0.19	competitive
17	7.35 ± 0.28	competitive
20	8.78 ± 0.39	competitive
22	2.45 ± 0.05	competitive

*K_i_ values determined with SigmaPlot at 3 different substrate concentrations.

Values are averages of at least two independent experiments, each performed in duplicate.

**Figure 3:**
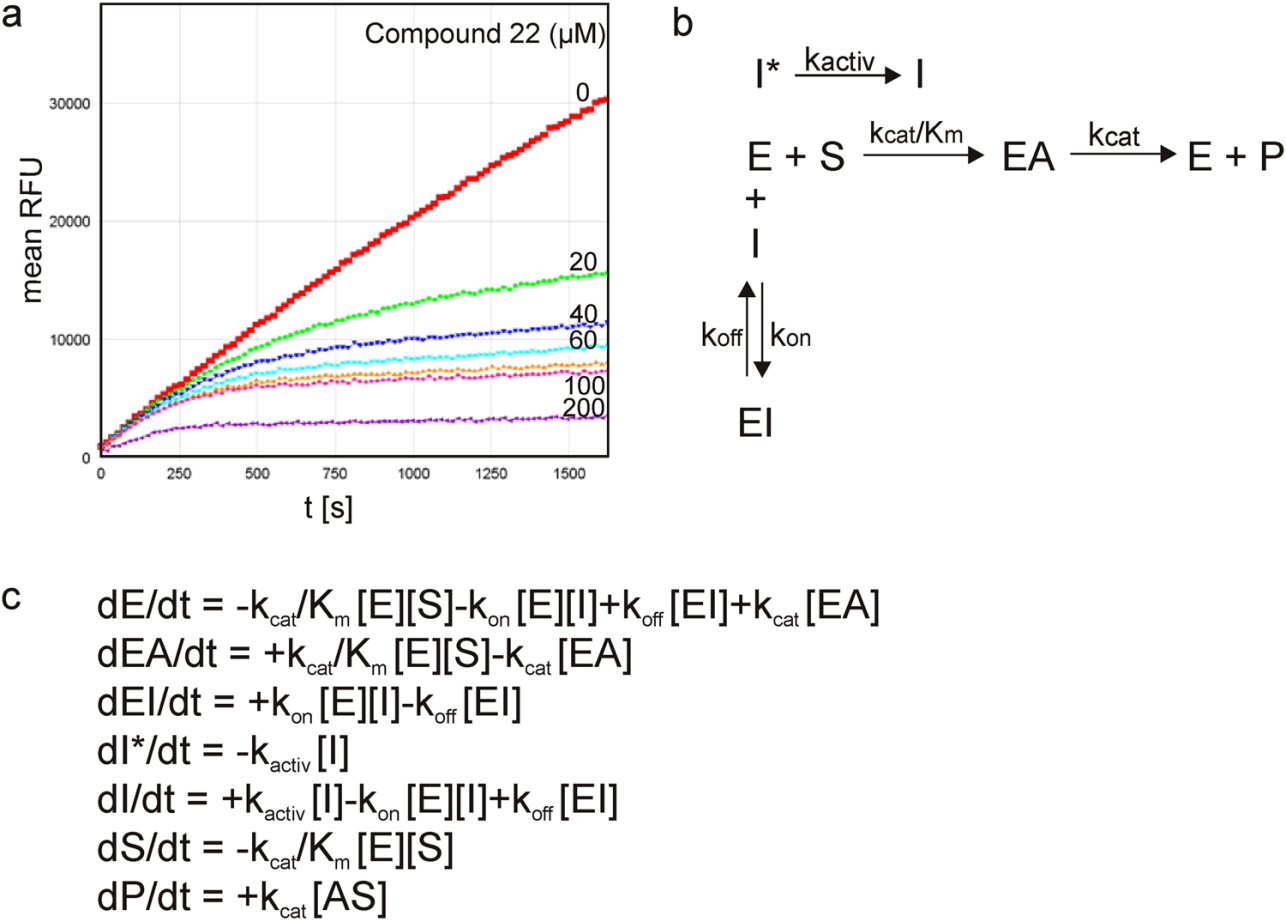
Kinetic evaluation of cathepsin X inhibitors. (**a**) Progress curves of the product formation by cathepsin X in the absence and presence of compound **22** at different concentrations of the compound (0 – 200 µM). (**b**) Reaction scheme describing the inhibition mechanism for the second group of compounds. (**c**) The system of differential equations for the reaction mechanism for the second group of inhibitors. k_cat_, K_m_, k_activ_, k_on_ and k_off_ are the kinetic constants, A is acylated substrate.

In-depth progress curves analysis of the second group of compounds points to the presence of an initial slow, rate limiting process (Fig. 3a). To distinguish between different possible mechanisms, recovery of enzyme activity was analyzed after rapid dilution of concentrated enzyme-inhibitor mixtures. Surprisingly, instantaneous reactivation of cathepsin X was observed after dilution. As immediate reactivation of enzyme is not compatible with any of the classical slow binding mechanisms, another mechanism had to be used for proper description of the inhibition (Fig. 3b). According to the proposed model, instantaneous competitive inhibition with preceding slow inhibitor isomerization describes the curves with the highest accuracy. The mechanism was analyzed using a system of differential equations (Fig. 3c). Non-linear regression analysis using ENZO software^20^ was used to determine the kinetic parameters. All five compounds from the second series exerted K_i_ values between 2 and 9 μM (Table 1), compound **22** being the most potent inhibitor (K_i_ of 2.45 ± 0.05 μM).

Based on their inhibitory potencies compounds **1**, **2**, **12**, **20** and **22** were chosen for further evaluation. First, their specificity towards cathepsin X relative to other related cathepsins was determined (Table 2). No inhibition of cathepsins L, H, S or cathepsin B endopeptidase activity was observed, while some of the compounds showed weak inhibition of cathepsin B exopeptidase activity (Table 2). Compounds **1**, **2** and **22** showed the highest specificity towards cathepsin X.

**Table 2:** K_i_ values against cathepsins B, L, H and S.

Compound	Cathepsin B (endopeptidase a.)	Cathepsin B (exopeptidase a.)	Cathepsin L	Cathepsin H	Cathepsin S
1	>1000	370.7 ± 97.9	974.9 ± 100.6	1872.9 ± 240.1	>1000
2	392.1 ± 121.1	210.3 ± 11.0	902.2 ± 89.7	483.1 ± 0.6	462.8 ± 42.9
12	>500	120.5 ± 0.9	>500	>500	n.i.
20	300.7 ± 213.7	83.1 ± 7.0	n.i.	>500	n.i.
22	310.4 ± 68.3	306.1 ± 38.1	n.i.	>1000	n.i.

n.i. No inhibition; K_i_ values determined with SigmaPlot at 3 different substrate concentrations.

Values are averages of at least two independent experiments, each performed in duplicate.

### Inhibitors 2, 20 and 22 affect the migration of PC-3 cells

**Figure 4:**
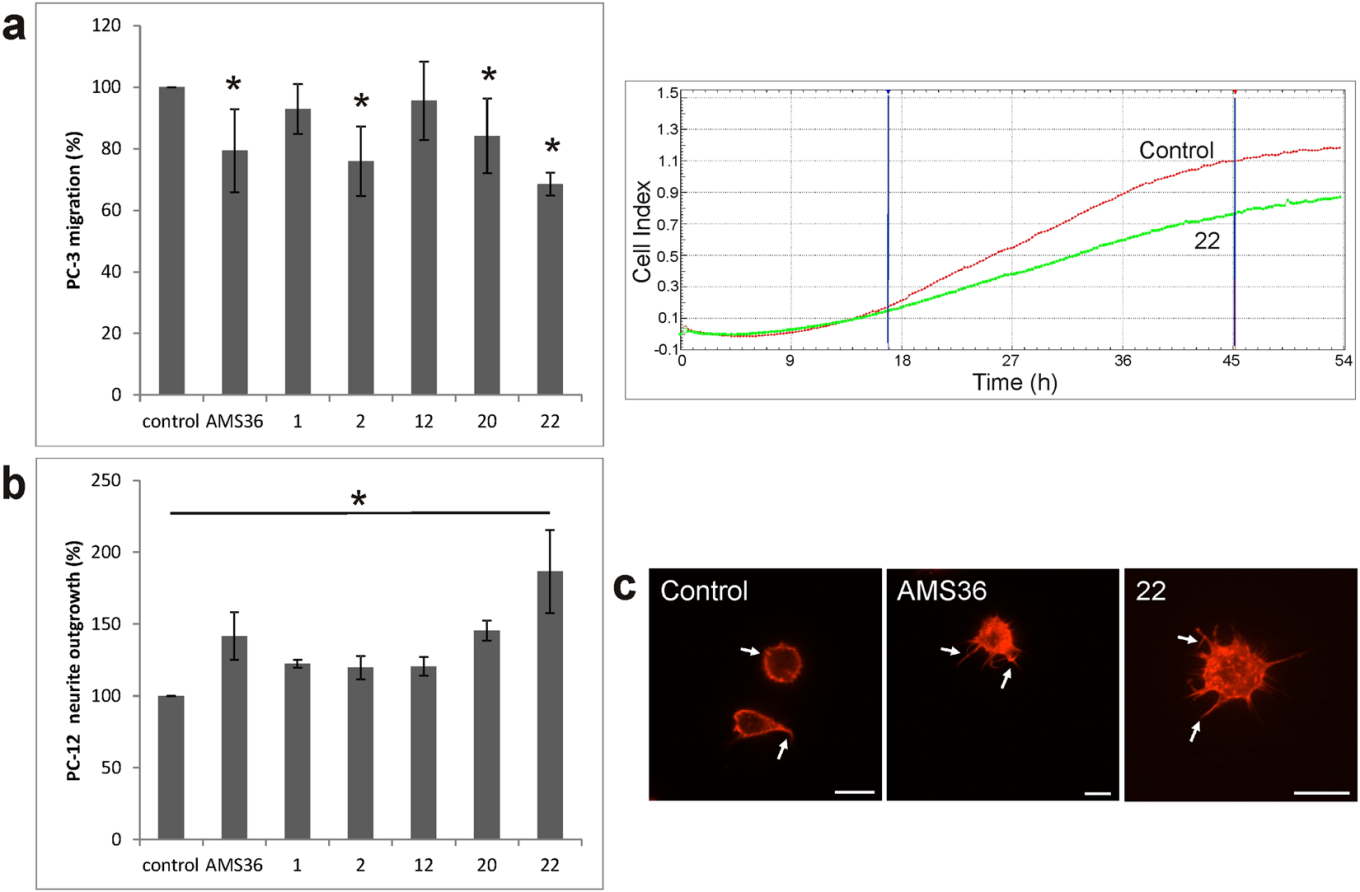
The new inhibitors affect cell processes associated with cathepsin X activity. (**a**) The level of PC-3 migration was assessed by increases in the curve slopes (1/h) that represent cell migration ability. Slopes were calculated from cell index values in the chosen time interval. Results are normalized to cells treated with DMSO (the control) with 100% migration ability. Cells treated with AMS36 served as a positive control. Right panel: representative migration curves of control and inhibitor **22** treated cells. The time interval used for calculating slopes is marked with blue lines. Assays were carried out three times, each in triplicate. Results are means ± S.E.M. *P ≤ 0.01 (T test). (**b**) The level of neurite outgrowth was determined after 48 hours of treatment with inhibitors. Results are expressed relative to the cell count and the control group (DMSO) was considered as 100% of neurite outgrowth. Values are means of two independent experiments, each performed in quadruplicate ± S.E.M. *P < 0.014 (T test). (**c**) Representative images of increased actin polymerization in the presence of cathepsin X inhibitors are shown using fluorescence microscopy and staining with TRITC-conjugated phalloidin. White arrows indicate rapid formation of neurites after 2-hour presence of cathepsin X inhibitors. Bars, 10 μm.

To confirm that the new inhibitors affect the pathological roles of cathepsin X in tumor cell migration and invasion^7, 16, 21^, the prostate cancer PC-3 cell line was used as a model system^16, 22^. Partition coefficient indicated that the compounds are able to enter cells (Supplementary Table S3) and for compound **22** intracellular presence was also determined by LC-MS/MS (Supplementary Table S4). Next, the compounds were tested for their cytotoxicity at 10 μM concentration over 24-hour and 48-hour periods. Inhibitors had no cytotoxic effects on PC-3 cells (Supplementary Fig. S2). Out of the 5 tested, compounds **2**, **20** and **22** showed 15–30% inhibition of PC-3 cell migration (Fig. 4a) in a standard migration assay using CIM plates and continuous compound presence in the upper and bottom compartments. Since cathepsin X also influences cell adhesion^16^ which is important in such experimental setting compound **22** was tested in additional migration assay. In this case the compound was not present in the bottom compartment and was added to the upper compartment only after cells had attached. In this way lower inhibition of cell migration was measured since in the bottom compartment there was no inhibitor to prevent the adhesion to the surface with electrodes (Supplementary Fig. S3). Interestingly, compounds **1** and **12**, which failed to affect cell migration, also showed trend toward less potent inhibition of cathepsin X enzyme activity in lysates of PC-3 cells than compounds **2**, **20** and **22** (Supplementary Fig. S2).

### Inhibitors affect neurite outgrowth in PC-12 cells

The pheochromocytoma PC-12 cell line was chosen as a model for the role of cathepsin X in neurite outgrowth, a process of neuronal differentiation. As for PC-3 cells, five selected compounds were first tested for cytotoxicity at 10 μM inhibitor concentration over 24-hour and 48-hour periods. Similarly, the inhibitors had no significant cytotoxic effects (Supplementary Fig. S2). However, besides compounds **2**, **20** and **22**, other inhibitors tested also showed a statistically significant increase of neurite outgrowth in PC-12 cells, **22** having the most pronounced effect of 85% increase (Fig. 4b). This is consistent with the inhibition of cathepsin X activity in PC-12 cell lysates where tested compounds inhibited cathepsin X, **22** again being the most potent (Supplementary Fig. S2). Actin polymerization is a prerequisite for neuronal differentiation, therefore the effect of **22** on polymerization was also evaluated. As shown by fluorescence microscopy, the down-regulation of neurite outgrowth by endogenous cathepsin X (control) was impaired by **22** (Fig. 4c).

## Discussion

There is increasing evidence showing that cathepsin X is involved in a variety of pathological processes leading to neurodegeneration, progression of cancer and other diseases. Excessive proteolytic activity cannot be balanced by endogenous inhibitors, and the number of exogenous inhibitors that could be used as potential drugs is small. To validate cathepsin X as a target for the treatment of neurodegeneration or cancer, the development of potent, selective, reversible and non-toxic inhibitors is urgently needed. In this study we have characterized triazole-based inhibitors of cathepsin X, which could be used as a molecular tool to study the role of cathepsin X in pathological states, and could even be useful in clinical practice. Their ability to impair tumor cell migration and to enhance neuritogenesis, two processes that are closely related to cathepsin X activity, additionally strengthens their potential for further *in vivo* application.

Among neurodegenerative disorders, cathepsin X is associated with Alzheimer’s disease, Parkinson’s disease, amyotrophic lateral sclerosis and Huntington’s disease^9^. It was demonstrated that, in brain tissue cathepsin X is present not only in neurons, but also in glial cells, and is involved in cell-cell interactions and various processes altering normal neuronal functions. For example, cathepsin X cleaves γ-enolase, which consequently eliminates its neurotrophic activity. Cathepsin X is also involved in the production of plasmin, regulation of neuronal differentiation and length of neurites^23^. Additionally, it can regulate neuronal survival by promoting apoptosis^24^, which is responsible for dopamine induced neuron death. We showed that down-regulation of cathepsin X expression or enzymatic activity by using siRNA silencing or AMS36 inhibitor, significantly attenuated neurotoxin induced neurodegeneration^24^. Cathepsin X has also been proposed to play an important role in cancer development and progression^3^. Unlike the related cathepsins B, L, S, V and K, which promote tumor cell migration and invasion through degradation of extracellular matrix^25^, cathepsin X contributes to tumor progression by proteolytic cleavage of other targets involved predominantly in cell adhesion and modulation of the cytoskeleton. Moreover, cathepsin X acts independently of proteolytic activity via the RGD motif present in the prodomain. By cleaving the C-terminal Tyr139 of profilin 1, a known tumor suppressor, cathepsin X can affect actin polymerization^22^ and increase tumor cell migration and invasion^16, 22^. It can also cleave the C-terminus of the β_2_-chain of integrin receptors and thus modulate adhesion of tumor cells to the extracellular matrix^26^. The latter can also be accomplished by the RGD motif, which interferes with the interactions between the RGD binding integrins and extracellular matrix proteins^21^. The fact that cathepsin X is secreted from tumor cells, as well as from tumor associated macrophages, predominantly as the inactive precursor form, supports RGD related functions^21^. The relevance of cathepsin X in tumor progression has been confirmed in several clinical studies, providing the association of higher levels of its precursor or active forms with shorter overall survival of cancer patients^8, 10, 27^.

There are several ways to influence the pathological processes involving cathepsin X in neurodegeneration and tumor progression and, among them, peptidase inhibitors represent the most effective pharmacological tool. Numerous peptidase inhibitors are already in clinical use^28, 29^ however, this is not the case for the inhibitors of cysteine cathepsins. Inhibitors of cathepsin K have received a lot of attention and many of them reached clinical trials, particularly for the treatment of osteoporosis. A large number of synthetic inhibitors have been developed for cathepsin B but far fewer for other cysteine cathepsins^28^. The first generations of cathepsin B inhibitors were irreversible inhibitors, which form a covalent bond with the catalytic cysteine. Since these inhibitors possess a reactive electrophilic group - a warhead - they can also bind to nucleophiles in off-target peptidases, which may lead to nonspecific inhibition and adverse side effects^29, 30^. Development has therefore been focused also on reversibly acting compounds. The cathepsin B inhibitors developed in our group, including 6-substituted 4-benzylthio-1,3,5-triazin-2(1 *H*)-ones^31^, nitroxoline and its derivatives^32–34^, and quinolone complexes with ruthenium^35^, have all provided very potent antitumor effects *in vitro* and *in vivo*. However, after a time their effectiveness has decreased and delayed overexpression of cathepsin X was proposed to be the main factor of this resistance, which compensates the tumor associated activity of cathepsin B^32, 36^. To improve antitumor therapy, cathepsin X inhibitors should be applied in parallel to those of cathepsin B to impair the renewed ability of tumor cells to migrate and invade.

The triazole ring is often used as a pharmacophore in medicinal chemistry^37^. Triazole-based aldehydes have been described as reversible inhibitors of cathepsins B, S, and H^38, 39^. As shown in our study, triazols also inhibit cathepsin X, and thus represent an effective tool to impair its excessive activity in pathological states. After initial screening, triazole derivatives with 2,3-dihydrobenzo[*b*][1,4]dioxine as an R^1^ substituent were identified as the most potent inhibitors of cathepsin X (Supplementary Table S1). Replacement of this substituent with substituted phenyl rings lowered the inhibitory potencies. The same effect was observed when a methyl fragment was introduced to the α-position of the carbonyl group (compounds **15** and **18**). The amide derivative **7** was also devoid of any inhibitory activity. The substitution pattern of the triazole ring did not affect the inhibitory potency to a significant extent. For example, the relative inhibition of compound **11** without any substituents was in the same range as that for compound **14** that has isopropyl and pyrrolidine substituents attached to the triazole. The best result was obtained with compound **22** that has an isopropyl fragment as R^4^. To test the theoretical probability of compound **22** binding to the cathepsin X active site, we performed docking experiment using AUTODOCK-Vina software^40^. The result shows possible formation of several intermolecular hydrogen bonds (Supplementary Fig. S4). New triazole-based inhibitors are also highly selective towards cathepsin X over other related cysteine cathepsins. For example, **22** inhibits cathepsin X with more than 100-fold lower values of K_i_ than those of the inhibition constants for exopeptidase or endopeptidase activity of cathepsin B. For other cathepsins the difference is even greater. Compound **22** also showed better selectivity for cathepsin X in cell lysates when both cathepsins were labeled with activity-based probe DCG-04 (Supplementary Fig. S5). Compound **22** has been shown to reduce the migration of PC-3 tumor cells, a process that is associated with increased cathepsin X activity. In addition to cell migration, cathepsin X is involved in several other processes associated with the progression of malignant diseases, namely epithelial-mesenchymal transition and bypassing of cellular senescence^3^. **22** and other triazole-based inhibitors are therefore promising hit compounds for development of antitumor agents. Moreover, they could also be used to enhance the effectiveness of antitumor activity of cathepsin B inhibitors, impairing the delayed compensatory effect of cathepsin X, as discussed above.

The effect of new cathepsin X inhibitors is also pronounced in the PC-12 cell model of neurite outgrowth. In these cells, cathepsin X significantly reduces neuritogenesis, presumably by impairing the neurotrophic activity of gamma enolase^23^. Triazole-based inhibitor **22** was the most effective promoter of neurite outgrowth. As in the case of migration, the effect was even greater than that of the irreversible inhibitor AMS36. Further, the neuroprotective role of **22** is also evident from the increased actin polymerization, an essential mechanism for neuronal differentiation. For this reason, new triazole-based inhibitors are also candidates for diminishing the activity of cathepsin X that is involved in neurodegenerative processes.

In conclusion, we have demonstrated that triazole derivatives are reversible, potent and selective inhibitors of cathepsin X, a peptidase identified as a target for the treatment of cancer and neurodegenerative diseases. In further studies the *in vivo* activity should be addressed to establish their potential as possible therapeutic agents.

## Reagents and Methods

### Compound characterization

Cathepsin X inhibitors were selected from the in-house compound library or obtained from Enamine Ltd (Kiev, Ukraine) and ChemBridge (ChemBridge Corporation, San Diego, CA, USA). Reagents and solvents used were obtained from Acros Organics, Sigma and Merck. 1 H NMR and 13 C spectra were recorded at 400 MHz and 100 MHz, respectively on a Bruker Avance III NMR spectrometer (Bruker Corporation, Billerica, MA, USA) at 295 K. Chemical shifts (δ) are reported in parts per million (ppm) and are referenced to the deuterated solvent used. High-resolution mass measurements were performed on a VG Analytical Autospec Q Micromass mass spectrometer (Fisons, VG Analytical, Manchester, UK) at the Jozef Stefan Institute, Ljubljana, Slovenia. Analytical reversed-phase HPLC analyses were performed on an Agilent 1100 LC modular system (Agilent Technologies, Santa Clara, CA, USA) equipped with a G1365B UV-Vis detector, a G1316A thermostat, a G1313A auto-sampler, and a ChemStation data system. An Agilent Eclipse Plus C18 column (4.6 × 150 mm, 5 µm), thermostated at 25 °C, was used with a flow rate of 1.0 mL/min. The detector was set to 254 nm.

#### HPLC method A

The sample solution (15 µL, 0.1 mg/mL in acetonitrile) was injected and eluted using a linear gradient of mobile phase A (0.1% TFA [v/v] in water) and mobile phase B (MeCN). The gradient (for mobile phase B) was: 0–15 min, 10–90%; 15–21 min, 90%; 21–23 min, 90–10%.

#### HPLC method B

The sample solution (15 µL, 0.1–0.5 mg/mL in acetonitrile, 20% DMSO) was injected and eluted using a linear gradient of mobile phase A (20 mM phosphate buffer, pH 6.95) and mobile phase B (MeCN). The gradient (for mobile phase B) was: 0–5 min, 5%; 5–15 min, 5–70%; 15–25 min, 70%; 25–30 min, 70–5%.

The purity of active compounds was determined by HPLC to be ≥95%.

#### Analytical data

2-((5-Amino-1*H*-1,2,4-triazol-3-yl)thio)-1-(2-methyl-1*H*-indol-3-yl)ethanone (**1**). ^1^H NMR (400 MHz, DMSO-*d*_6_) δ 2.71 (s, 3 H), 4.59 (s, 2 H), 6.06 (s, 2 H), 7.14–7.19 (m, 2 H), 7.37–7.40 (m, 1 H), 7.97–7.99 (m, 1 H), 11.89 (br s, 1 H), 11.99 (br s, 1 H); HRMS (ESI) m/z calculated for C_13_H_14_N_5_OS [M + H]^+^ 288.0919, found 288.0911; purity by HPLC (*Method B*, *t*_R_ = 13.53 min): 97%.

2-((5-Amino-1*H*-1,2,4-triazol-3-yl)thio)-1-(2,3-dihydrobenzo[*b*][1,4]dioxin-6-yl)ethanone (**2**). ^1^H NMR (400 MHz, DMSO-*d*_6_) δ 4.28–2.30 (m, 2 H), 4.32–2.34 (m, 2 H), 4.53 (s, 2 H), 6.06 (s, 2 H), 6.98 (d, *J* = 8.5 Hz, 1 H), 7.47 (d, *J* = 2.1 Hz, 1 H), 7.52 (dd, *J*_1_ = 8.5 Hz, *J*_2_ = 2.1 Hz, 1 H), 11.90 (br s, 1 H); HRMS (ESI) m/z calculated for C_12_H_13_N_4_O_3_S [M + H]^+^ 293.0708, found 293.0712; purity by HPLC (*Method B*, *t*_R_ = 13.62 min): 95%.

5-(2-Aminoethoxy)-2-chloro-*N*-(3-cyano-4,5,6,7-tetrahydrobenzo[*b*] thiophen-2-yl)benzamide (**3**). ^1^H NMR (400 MHz, DMSO-*d*_6_) δ 1.75–1.80 (m, 4 H), 2.50–2.55 (m, 2 H), 2.63 (t, *J* = 4.8 Hz, 2 H), 3.24 (t, *J* = 5.2 Hz, 2 H), 4.22 (t, *J* = 7.0 Hz, 2 H), 7.16 (dd, *J*_1_ = 8.8, *J*_2_ = 3.1 Hz, 1 H), 7.21 (d, *J* = 3.1 Hz, 1 H), 7.51 (d, *J* = 8.7, 1 H); HRMS (ESI) m/z calculated for C_18_H_19_ClN_3_O_2_S [M + H]^+^ 376.0887, found 376.0875; purity by HPLC (*Method A*, *t*_R_ = 13.02 min): 95%.

5-(3-Aminopropoxy)-2-chloro-*N*-(3-cyano-4,5,6,7-tetrahydrobenzo[*b*] thiophen-2-yl)benzamide (**4**). ^1^H NMR (400 MHz, CDCl_3_) δ 1.59–2.44 (br s, 2 H), 1.83–1.90 (m, 4 H), 1.94 (q, *J* = 6.5 Hz, 2 H), 2.62–2.73 (m, 4 H), 2.85 (t, *J* = 6.8 Hz, 2 H), 4.08 (t, *J* = 6.1 Hz, 2 H), 7.02 (dd, *J*_1_ = 8.8, *J*_2_ = 3.1 Hz, 1 H), 7.39 (d, *J* = 8.8 Hz, 1 H), 7.46 (d, *J* = 3.1 Hz, 1 H); HRMS (ESI) m/z calculated for C_19_H_21_ClN_3_O_2_S [M + H]^+^ 390.1043, found 390.1047; purity by HPLC (*Method A*, *t*_R_ = 14.13 min): 99%.

*N*-1,4-dioxooctahydropyrrolo[1,2-*a*]pyrazin-7-yl)-1,8-naphthyridine-2-carboxamide (**5**). ^1^H NMR (400 MHz, DMSO-*d*_6_) δ 2.22–2.30 (m, 1 H), 2.40–2.46 (m, 1 H), 3.51–3.58 (m, 2 H), 3.69 (dd, *J*_1_ = 11.4 Hz, *J*_2_ = 8.3 Hz, 1 H), 4.03 (dd, *J*_1_ = 16.3 Hz, *J*_2_ = 1.1 Hz, 1 H), 4.35–4.39 (m, 1 H), 4.69–4.79 (m, 1 H), 7.75 (dd, *J*_1_ = 8.2 Hz, *J*_2_ = 4.2 Hz, 1 H), 8.17 (d, *J* = 4.4 Hz, 1 H), 8.26 (d, *J* = 8.3 Hz, 1 H), 8.59 (dd, *J*_1_ = 8.2 Hz, *J*_2_ = 2.0 Hz, 1 H), 8.69 (d, *J* = 8.4 Hz, 1 H), 9.21 (dd, *J*_1_ = 4.2 Hz, *J*_2_ = 2.0 Hz, 1 H), 9.24 (d, *J* = 8.1 Hz, 1 H); HRMS (ESI) m/z calculated for C_16_H_16_N_5_O_3_ [M + H]^+^ 326.1253, found 326.1251; purity by HPLC (*Method B*, *t*_R_ = 11.62 min): 98%.

2-((4*H*-1,2,4-triazol-3-yl)thio)-1-(2-methyl-1*H*-indol-3-yl)ethanone (**6**). ^1^H NMR (400 MHz, DMSO-*d*_6_) δ 2.72 (s, 3 H), 4.76 (br s, 2 H), 7.15–7.19 (m, 2 H), 7.39–7.41 (m, 1 H), 7.99–8.01 (m, 1 H), 8.54 (br s, 1 H), 12.02 (br s, 1 H), 14.02 (br s, 1 H); HRMS (ESI) m/z calculated for C_13_H_13_N_4_OS [M + H]^+^ 273.0810, found 273.0813; purity by HPLC (*Method B*, *t*_R_ = 14.13 min): 99%.

2-((5-Amino-1*H*-1,2,4-triazol-3-yl)thio)-1-(6,7-dimethoxy-3,4-dihydro isoquinolin-2(1 *H*)-yl)ethanone (**7**). ^1^H NMR (400 MHz, DMSO-*d*_6_) δ 2.66–2.69 (m, 1 H), 2.78 (t, *J* = 5.7 Hz, 1 H), 3.64 (t, *J* = 6.0 Hz, 1 H), 3.68 (t, *J* = 5.9 Hz, 1 H), 3.71 (s, 3 H), 3.71 (s, 3 H), 4.09 (br s, 2 H), 4.51 (br s, 1.2 H), 4.62 (br s, 0.8 H), 6.07 (br s, 2 H), 6.74–6.79 (m, 2 H), 11.94 (br s, 1 H); HRMS (ESI) m/z calculated for C_15_H_20_N_5_O_3_S [M + H]^+^ 350.1287, found 350.1283; purity by HPLC (*Method B*, *t*_R_ = 13.03 min): 97%.

2-((5-Amino-1*H*-1,2,4-triazol-3-yl)thio)-1-(benzo[*d*][1,3]dioxol-5-yl) ethanone (**8**). ^1^H NMR (400 MHz, DMSO-*d*_6_) δ 4.54 (s, 2 H), 6.06 (br s, 2 H), 6.15 (br s, 2 H), 7.05 (d, *J* = 8.2 Hz, 1 H), 7.46 (d, *J* = 1.7 Hz, 1 H), 7.64 (dd, *J*_1_ = 8.2 Hz, *J*_2_ = 1.8 Hz, 1 H), 11.90 (br s, 1 H); HRMS (ESI) m/z calculated for C_11_H_11_N_4_O_3_S [M + H]^+^ 279.0552, found 279.0555; purity by HPLC (*Method B*, *t*_R_ = 13.52 min): 96%.

2-((5-Amino-1*H*-1,2,4-triazol-3-yl)thio)-1-(4-phenoxyphenyl)ethanone (**9**). ^1^H NMR (400 MHz, DMSO-*d*_6_) δ 4.59 (s, 2 H), 6.06 (br s, 2 H), 7.03–7.04 (m, 1 H), 7.06–7.07 (m, 1 H), 7.13–7.14 (m, 1 H), 7.15–7.16 (m, 1 H), 7.24–7.28 (m, 1 H), 7.45–7.50 (m, 2 H), 8.00–8.04 (m, 2 H), 11.91 (br s, 1 H); HRMS (ESI) m/z calculated for C_16_H_15_N_4_O_2_S [M + H]^+^ 327.0916, found 327.0913; purity by HPLC (*Method B*, *t*_R_ = 16.28 min): 96%.

1-([1,1′-Biphenyl]-4-yl)-2-((5-amino-1*H*-1,2,4-triazol-3-yl)thio)ethanone (**10**). ^1^H NMR (400 MHz, DMSO-*d*_6_) δ 4.66 (s, 2 H), 6.07 (br s, 2 H), 7.42–7.46 (m, 1 H), 7.50–7.54 (m, 2 H), 7.75–7.78 (m, 2 H), 7.83–7.86 (m, 2 H), 8.06–8.09 (m, 2 H), 11.92 (br s, 1 H); HRMS (ESI) m/z calculated for C_16_H_15_N_4_OS [M + H]^+^ 311.0967, found 311.0961; purity by HPLC (*Method B*, *t*_R_ = 16.12 min): 98%.

2-((1*H*-1,2,4-triazol-3-yl)thio)-1-(2,3-dihydrobenzo[*b*][1,4]dioxin-6-yl) ethanone (**11**). ^1^H NMR (400 MHz, DMSO-*d*_6_) δ 4.28–4.35 (m, 4 H), 4.71 (s, 2 H), 6.99 (d, *J* = 8.4 Hz, 1 H), 7.50 (d, *J* = 2.1 Hz, 1 H), 7.54 (dd, *J*_1_ = 8.5 Hz, *J*_2_ = 2.2 Hz, 1 H), 8.47 (br s, 1 H), 14.00 (br s, 1 H); HRMS (ESI) m/z calculated for C_12_H_12_N_3_O_3_S [M + H]^+^ 278.0599, found 278.0604; purity by HPLC (*Method B*, *t*_R_ = 14.21 min): 95%.

1-(2,3-Dihydrobenzo[*b*][1,4]dioxin-6-yl)-2-((1-methyl-1*H*-1,2,4-triazol-3-yl)thio)ethanone (**12**). ^1^H NMR (400 MHz, DMSO-*d*_6_) δ 3.59 (s, 3 H), 4.28–4.35 (m, 4 H), 4.75 (s, 2 H), 6.99 (d, *J* = 8.4 Hz, 1 H), 7.50 (d, *J* = 2.1 Hz, 1 H), 7.53 (dd, *J*_1_ = 8.4 Hz, *J*_2_ = 2.2 Hz, 1 H), 8.53 (s, 1 H); HRMS (ESI) m/z calculated for C_13_H_14_N_3_O_3_S [M + H]^+^ 292.0756, found 292.0757; purity by HPLC (*Method B*, *t*_R_ = 14.20 min): 97%.

2-((5-Amino-1*H*-1,2,4-triazol-3-yl)thio)-1-(*p*-tolyl)ethanone (**13**). ^1^H NMR (400 MHz, DMSO-*d*_6_) δ 2.38 (s, 3 H), 4.60 (s, 2 H), 6.06 (br s, 2 H), 7.34 (d, *J* = 8.0 Hz, 2 H), 7.89 (d, *J* = 8.2 Hz, 2 H), 11.90 (br s, 1 H); HRMS (ESI) m/z calculated for C_11_H_13_N_4_OS [M + H]^+^ 249.0810, found 249.0806; purity by HPLC (*Method B*, *t*_R_ = 14.28 min): 99%.

2-((4-Cyclopropyl-5-(pyrrolidin-1-yl)-4*H*-1,2,4-triazol-3-yl)thio)-1-(2,3-dihydrobenzo[*b*][1,4]dioxin-6-yl)ethanone (**14**). ^1^H NMR (400 MHz, DMSO-*d*_6_) δ 0.92–0.96 (m, 2 H), 1.06–1.11 (m, 2 H), 1.84–1.88 (m, 4 H), 3.11–3.17 (m, 1 H), 3.37–3.40 (m, 4 H), 4.28–4.35 (m, 4 H), 4.69 (s, 2 H), 6.99 (d, *J* = 8.4 Hz, 1 H), 7.50 (d, *J* = 2.0 Hz, 1 H), 7.53 (dd, *J*_1_ = 8.4 Hz, *J*_2_ = 2.2 Hz, 1 H); HRMS (ESI) m/z calculated for C_19_H_23_N_4_O_3_S [M + H]^+^ 387.1491, found 387.1499; purity by HPLC (*Method B*, *t*_R_ = 16.73 min): 98%.

2-((5-Amino-1*H*-1,2,4-triazol-3-yl)thio)-1-(2-methyl-1*H*-indol-3-yl)propan-1-one (**15**). ^1^H NMR (400 MHz, DMSO-*d*_6_) δ 1.57 (d, *J* = 6.7 Hz, 3 H), 2.71 (s, 3 H), 5.13 (q, *J* = 6.7 Hz, 1 H), 6.12 (s, 2 H), 7.12–7.18 (m, 2 H), 7.37–7.41 (m, 1 H), 7.95–7.99 (m, 1 H), 12.00 (br s, 1 H), 12.05 (br s, 1 H); HRMS (ESI) m/z calculated for C_14_H_16_N_5_OS [M + H]^+^ 302.1076, found 302.1071; purity by HPLC (*Method B*, *t*_R_ = 13.91 min): 96%.

4-(2-((5-Amino-1*H*-1,2,4-triazol-3-yl)thio)acetyl)benzonitrile (**16**).^1^H NMR (400 MHz, DMSO-*d*_6_) δ 4.64 (s, 2 H), 6.06 (s, 2 H), 8.01–8.04 (m, 2 H), 8.12–8.14 (m, 2 H), 11.92 (br s, 1 H); HRMS (ESI) m/z calculated for C_11_H_10_N_5_OS [M + H]^+^ 260.0606, found 260.0599; purity by HPLC (*Method B*, *t*_R_ = 13.18 min): 95%.

2-((3-Cyclopropyl-1*H*-1,2,4-triazol-5-yl)thio)-1-(2,3-dihydrobenzo[*b*][1,4] dioxin-6-yl)ethan-1-one (**17**). ^1^H NMR (400 MHz, DMSO-*d*_6_) δ 0.78–0.82 (m, 2 H), 0.93–0.96 (m, 2 H), 1.90–1.97 (m, 1 H), 4.28–4.30 (m, 2 H), 4.33–4.35 (m, 2 H), 4.60 (s, 2 H), 6.98 (d, *J* = 8.4 Hz, 1 H), 7.49 (d, *J* = 2.0 Hz, 1 H), 7.52 (dd, *J*_1_ = 8.4 Hz, *J*_2_ = 2.2 Hz, 1 H), resonance for NH missing; HRMS (ESI) m/z calculated for C_15_H_16_N_3_O_3_S [M + H]^+^ 318.0912, found 318.0915; purity by HPLC (*Method B*, *t*_R_ = 15.44 min): 96%.

1-(3,4-Dimethoxyphenyl)-2-((4-methyl-4*H*-1,2,4-triazol-3-yl)thio)propan-1-one (**18**). ^1^H NMR (400 MHz, DMSO-*d*_6_) δ 1.49 (d, *J* = 6.9 Hz, 3 H), 3.43 (s, 3 H), 3.80 (s, 3 H), 3.86 (s, 3 H), 5.30 (d, *J* = 6.9 Hz, 1 H), 7.07 (d, *J* = 8.6 Hz, 1 H), 7.46 (d, *J* = 2.1 Hz, 1 H), 7.69 (dd, *J*_1_ = 8.6 Hz, *J*_2_ = 2.1 Hz, 1 H), 8.58 (s, 1 H); HRMS (ESI) m/z calculated for C_14_H_18_N_3_O_3_S [M + H]^+^ 308.1069, found 308.1063; purity by HPLC (*Method B*, *t*_R_ = 14.22 min): 95%.

2-((4,6-Diaminopyrimidin-2-yl)thio)-1-(2,3-dihydrobenzo[*b*][1,4]dioxin-6-yl)ethan-1-one (**19**). ^1^H NMR (400 MHz, DMSO-*d*_6_) δ 4.27–4.29 (m, 2 H), 4.32–4.34 (m, 2 H), 4.54 (s, 2 H), 5.13 (s, 1 H), 6.10 (br s, 4 H), 6.98 (d, *J* = 8.5 Hz, 1 H), 7.50 (d, *J* = 2.1 Hz, 1 H), 7.57 (dd, *J*_1_ = 8.5 Hz, *J*_2_ = 2.2 Hz, 1 H); HRMS (ESI) m/z calculated for C_14_H_15_N_4_O_3_S [M + H]^+^ 319.0865, found 319.0862; purity by HPLC (*Method B*, *t*_R_ = 14.79 min): 95%.

1-(2,3-Dihydrobenzo[*b*][1,4]dioxin-6-yl)-2-((4-(*o*-tolyl)-4*H*-1,2,4-triazol-3-yl)thio)ethan-1-one (**20**). ^1^H NMR (400 MHz, DMSO-*d*_6_) δ 2.07 (s, 3 H), 4.29–4.31 (m, 2 H), 4.33–4.35 (m, 2 H), 4.82 (s, 2 H), 7.00 (d, *J* = 8.5 Hz, 1 H), 7.35–7.43 (m, 2 H), 7.48–7.51 (m, 3 H), 7.53 (dd, *J*_1_ = 8.5 Hz, *J*_2_ = 2.2 Hz, 1 H), 8.77 (s, 1 H); HRMS (ESI) m/z calculated for C_19_H_18_N_3_O_3_S [M + H]^+^ 368.1069, found 368.1059; purity by HPLC (*Method B*, *t*_R_ = 16.79 min): 98%.

2-((5-Amino-1*H*-1,2,4-triazol-3-yl)thio)-1-(3-fluoro-4-methoxyphenyl)ethan-1-one (**21**).^1^H NMR (400 MHz, DMSO-*d*_6_) δ 3.94 (s, 3 H), 4.57 (s, 2 H), 6.06 (s, 2 H), 7.30 (t, *J* = 8.6 Hz, 1 H), 7.67 (dd, *J*_1_ = 12.2 Hz, *J*_2_ = 2.1 Hz, 1 H), 7.67 (ddd, *J*_1_ = 8.6 Hz, *J*_2_ = 2.1 Hz, *J*_3_ = 1.0 Hz, 1 H), 11.92 (br s, 1 H); HRMS (ESI) m/z calculated for C_11_H_12_FN_4_O_2_S [M + H]^+^ 283.0665, found 283.0667; purity by HPLC (*Method B*, *t*_R_ = 14.04 min): 95%.

1-(2,3-Dihydrobenzo[*b*][1,4]dioxin-6-yl)-2-((4-isopropyl-4*H*-1,2,4-triazol-3-yl)thio)ethan-1-one (**22**). ^1^H NMR (400 MHz, DMSO-*d*_6_) δ 1.40 (d, *J* = 6.7 Hz, 6 H), 4.28–4.30 (m, 2 H), 4.33–4.35 (m, 2 H), 4.39 (p, *J* = 7.8 Hz, 1 H), 4.79 (s, 2 H), 6.99 (d, *J* = 8.4 Hz, 1 H), 7.50 (d, *J* = 2.1 Hz, 1 H), 7.53 (dd, *J*_1_ = 8.4 Hz, *J*_2_ = 2.2 Hz, 1 H), 8.72 (s, 1 H); HRMS (ESI) m/z calculated for C_15_H_18_N_3_O_3_S [M + H]^+^ 320.1069, found 320.1064; purity by HPLC (*Method B*, *t*_R_ = 15.44 min): 96%.

1-(2,3-Dihydrobenzo[*b*][1,4]dioxin-6-yl)-2-((4-phenyl-4*H*-1,2,4-triazol-3-yl)thio)ethan-1-one (**23**).^1^H NMR (400 MHz, DMSO-*d*_6_) δ 4.29–4.31 (m, 2 H), 4.33–4.36 (m, 2 H), 4.85 (s, 2 H), 7.00 (d, *J* = 8.5 Hz, 1 H), 7.49 (d, *J* = 2.0 Hz, 1 H), 7.52–7.63 (m, 6 H), 8.86 (s, 1 H); HRMS (ESI) m/z calculated for C_18_H_16_N_3_O_3_S [M + H]^+^ 354.0912, found 354.0920; purity by HPLC (*Method B*, *t*_R_ = 15.60 min): 97%.

1-(2,3-Dihydrobenzo[*b*][1,4]dioxin-6-yl)-2-((4-(2-methoxyphenyl)-4*H*-1,2,4-triazol-3-yl)thio)ethan-1-one (**24**). ^1^H NMR (400 MHz, DMSO-*d*_6_) δ 3.80 (s, 3 H), 4.29–4.30 (m, 2 H), 4.33–4.35 (m, 2 H), 4.78 (s, 2 H), 6.99 (d, *J* = 8.4 Hz, 1 H), 7.12 (dt, *J*_1_ = 7.6 Hz, *J*_2_ = 1.2 Hz, 1 H), 7.29 (dd, *J*_1_ = 8.4 Hz, *J*_2_ = 1.2 Hz, 1 H), 7.40 (dd, *J*_1_ = 7.8 Hz, *J*_2_ = 1.6 Hz, 1 H), 7.48 (d, *J* = 2.0 Hz, 1 H), 7.50–7.57 (m, 2 H), 8.69 (s, 1 H); HRMS (ESI) m/z calculated for C_19_H_18_N_3_O_4_S [M + H]^+^ 384.1018, found 384.1011; purity by HPLC (*Method B*, *t*_R_ = 16.66 min): 96%.

2-((5-Amino-1*H*-1,2,4-triazol-3-yl)thio)-1-(2-methoxyphenyl)ethan-1-one (**25**). ^1^H NMR (400 MHz, DMSO-*d*_6_) δ 3.90 (s, 3 H), 4.45 (s, 2 H), 6.03 (s, 2 H), 7.02–7.06 (m, 1 H), 7.19 (dd, *J*_1_ = 8.4 Hz, *J*_2_ = 0.8 Hz, 1 H), 7.55–7.61 (m, 2 H), 11.88 (br s, 1 H); HRMS (ESI) m/z calculated for C_11_H_13_N_4_O_2_S [M + H]^+^ 265.0759, found 265.0756; purity by HPLC (*Method B*, *t*_R_ = 13.76 min): 97%.

### Enzymes and assay buffers

Recombinant or native human cathepsins were used: recombinant cathepsin X and cathepsin S were expressed in *Pichia pastoris* ^41^, recombinant cathepsins B and L in *E*. *coli* ^42, 43^, native cathepsin H was isolated from human liver^44^. Assay buffers for determining activities of cathepsins X and L consisted of 100 mM sodium acetate buffer, pH 5.5, for cathepsins H, S and B (endopeptidase activity) of 100 mM phosphate buffer pH 6.8, 6.5 and 6.0, respectively and for cathepsin B (exopeptidase activity) of 60 mM acetate buffer pH 5.0. Each assay buffer contained 0.01% Triton X-100 to prevent false positive inhibition due to aggregation of compounds^45^, 5 mM cysteine, 1.5 mM EDTA and 0.1% PEG 8000. Enzymes were activated at 37 °C for 5 min before the assays.

### Determination of relative inhibition

Abz-FEK(Dnp)-OH was used as cathepsin X specific substrate in activity assays^46^. 90 μL of activated cathepsin X (20 nM) was added to a black 96-well plate with 5 μL of substrate (3.25 μM final concentration) and 5 μL of the compound (50 μM final concentration). The reaction was monitored continuously at 420 nm ± 10 nm with excitation at 320 nm ± 20 nm and at 37 °C. Relative inhibition (%) was calculated with the equation (1 − *v*_*i*_/*v*_0_) × 100, where *v*_*i*_ is reaction velocity in the presence of the compound and *v*_0_ is reaction velocity without the compound.

To detect cathepsin X activity in cell lysates, 95 μL of activated cell lysates (0.2 mg/mL total protein in assay buffer) were added to a black 96-well plate containing 5 μL of the substrate. The further procedure was the same as above. In some experiments lysates of non-treated cells were pre-incubated with 10 μM of compounds for 30 minutes and then activity was measured as described above.

### Determination of K_i_ values and inhibition mechanisms

K_i_ was determined by measuring the initial rate of hydrolysis at three different substrate concentrations (Supplementary Table S2) in the presence of seven different compound concentrations (0, 20, 40, 60, 80, 100 and 200 μM). 90 μL of the activated cathepsin was added to a black 96-well plate with 5 μL of the substrate and 5 μL of the compound. Formation of the fluorescent degradation product was monitored continuously at 460 nm ± 10 nm with excitation at 380 nm ± 20 nm (for AMC substrates) and, as noted above for Abz substrates, at 37 °C. All measurements were performed twice and in duplicate. The resulting data were analyzed by non-linear regression using the software SigmaPlot^®^, Enzyme Kinetics Module™ 1.3 and were fitted to the equation *v = v*_*max*_ *[S]/([S] (1 + [I]/αK*_*i*_ *) + K*_*m*_ *(1 + [I]/K*_*i*_ *))* (first group of compounds). For K_i_ determination against cathepsin X for the compounds from the second group, progress curves in the absence and presence of each putative inhibitor were fitted to the reaction mechanism presented in Fig. 3b. In this case the resulting data as well as reactivation curves were fitted to the equation system presented in Fig. 3c using ENZO application^20^. For calculation of kinetic parameters from reaction progress curves the fluorescence coefficient for conversion of arbitrary fluorescence units to concentration was first determined. The fluorescence coefficient was calculated as the slope from the plot of initial substrate concentration vs. height of the plateau of the progress curve after complete hydrolysis of increasing concentrations of substrate, using linear regression.

### Determination of the reversibility of binding

The reversibility of binding was determined in two separate experiments. In the washout experiment^47^ 20 nM cathepsin X was mixed with a saturating level of the inhibitor (100 μM) and incubated for 1 hour at room temperature. One half of the reaction mixture was assayed for activity and the other half was diluted approximately 200000-fold with assay buffer prior to the activity assay. Step-wise dilution and concentration was performed on a Centricon (Millipore) with 10 kDa cut-off. In the dilution method^19^ cathepsin X (5 μM) was incubated with inhibitor (100 μM). Half the reaction mixture was diluted 100-fold with the assay buffer containing substrate and immediately assayed for activity, the other half was incubated for 2 hours, diluted 100-fold and then assayed for activity.

### Cell cultures

Human prostate cancer cell line PC-3 cells (obtained from ATCC, Manassas, USA; ATCC Number: CRL-1435) were cultured in F-12 (Gibco) and Advanced DMEM (Gibco) (1:1) with 10% FBS, 1% penicillin/streptomycin and 1% L-glutamine. Rat pheochromocytoma PC-12 cells (obtained from ATCC, Manassas, USA; ATCC Number: CRL-1721) were cultured in Advanced DMEM (Gibco) with 5% FBS, 10% heat-inactivated horse serum, 1% penicillin/streptomycin and 1% L-glutamine.

For testing the enzyme activity in cell lysates, cells were treated with 10 μM of compounds. After 24 hours lysates were prepared in 50 mM Na acetate buffer pH 5.5 with 1 mM EDTA, 100 mM NaCl and 0.25% Triton X-100. Total protein concentration was determined by DC Protein Assay (Bio Rad) according to the instructions.

### Cytotoxicity assay

6.5 × 10^4^ PC-3 cells/ml were seeded in a 96-well plate. PC-12 cells were seeded on poly-L-lysine (Sigma) coated plates in serum-free medium at a concentration of 4 × 10^4^/well. Cells were treated with various concentrations of compounds (1, 2.5, 5 and 10 μM) for 24 or 48 hours. Cytotoxicity was determined with MTS reagent (CellTiter 96 Aqueous One Solution Cell Proliferation Assay, Promega) and A_492_ measured after 1 hour.

### Real time cell migration assay

Cell migration assay was performed on a Real-Time Cell Analyzer Dual Plate (RTCA DP) Instrument, xCELLigence System (ACEA Biosciences). CIM plates were coated with fibronectin (10 μg/ml, BD Biosciences) on the down and upper side of the microporous PET membrane for 30 min at room temperature and 2 hours at 37 °C, respectively. Excess fibronectin was removed and wells washed with phosphate buffer saline (PBS). Compounds (10 μM) were added to the complete media (lower chambers) or serum-free media (upper chambers). 0.1% DMSO was used as a control. 2 × 10^4^ cells were plated per well. In additional experimental setting, inhibitors were added only to the upper chambers 3 hours after experiment started in order to measure their impact only on the cell migration and not the adhesion. Impedance data, reported as cell index, were measured continuously every 15 min for 72 hours and then analyzed with the RTCA Software (ACEA Biosciences, Inc.). Results are normalized to cells treated with DMSO.

### Neurite outgrowth assay

PC-12 cells were seeded in 96-well plates (4 × 10^4^/well) coated with poly-L-lysine. Next day, the cells were treated with compounds (10 µM) in serum-free medium, and neurite outgrowth was measured after 48 h using Neurite Outgrowth Staining Kit (Molecular Probes), in accordance with the manufacturer’s instructions. Fluorescence intensity was measured at 525 nm with excitation at 483 nm for cell viability indicator and at 567 nm with excitation at 554 nm for cell membrane indicator. Results, expressing increased neurite outgrowth, were normalized to cells treated with DMSO.

### Determination of F-actin content of cells

For the fluorescence staining of F-actin, PC-12 cells were cultured on glass coverslips coated with poly-L-lysine (4 × 10^4^/ml). Next day, cells were treated with compounds (10 µM) in serum-free medium for 2 h. The cells were then fixed in 5% formalin (w/v) in PBS (pH 7.4) for 5 min and permeabilized with 0.1% Triton X-100 in PBS for 3 min. After washing with PBS, cells were incubated with phalloidin-TRITC (500 ng/mL) (Sigma) in 20 mM TrisHCl (pH 7.4) at 37 °C for 30 min. After additional washing with PBS, the Prolong Antifade kit was used for mounting the coverslips on glass slides. Fluorescence microscopy was performed using an Olympus IX 81 motorized inverted microscope.

## Electronic supplementary material

Supplementary Information
