# Supplementary material for: Identification and characterization of the novel reversible and selective cathepsin X inhibitors
Source: Sci Rep. 2017 Sep 13;7:11459. doi: 10.1038/s41598-017-11935-1 (PMC5597618; doi:10.1038/s41598-017-11935-1)
Supplement: Supplementary file 1 — Supplementary Information [file 41598_2017_11935_MOESM1_ESM.pdf]

Identification and characterization of the novel reversible and selective cathepsin X inhibitors

Urša Pečar Fonović, Ana Mitrović, Damijan Knez, Tanja Jakoš, Anja Pišlar, Boris Brus, Bojan Doljak, Jure Stojan, Simon Žakelj, Jurij Trontelj, Stanislav Gobec, Janko Kos

Supplementary Methods

Evaluation of cellular uptake and retention of compound 22: sample preparation and LC-MS/MS analysis

PC-3 cells were treated for 1 hour with compound **22** (50 μM). Cells were washed twice with cold PBS, trypsinized, washed once more and centrifuged. A pellet of cells was resuspended in 200 μL of PBS (or in 200 μL of PBS containing 5μM inhibitor for standard addition) and transferred to 1.5 mL Eppendorf vials prefilled with 0.1 mm and 0.5 mm glass beads (NextAdvance, USA). All cell samples were then homogenized with a BulletBlender (NextAdvance, USA) for 5 min at speed setting »6«. The homogenates were mixed with triple volume of ice-cold acetonitrile containing 1% formic acid and stored at -20 °C for 18 hours. The same protein precipitation procedure was applied to 300 μL samples of media sampled before and after the cellular uptake experiment. After centrifugation at 15000×g for 10 min at 4 °C the supernatants were transferred to vials for analysis by an Agilent 1290 Infinity LC coupled to an Agilent 6460 triple quadrupole mass spectrometer (Agilent Technologies, USA). 1 μL samples were injected onto a 50 × 2.1 mm, 2.6 μm Kinetex C18 column (Phenomenex) maintained at 37 °C and eluted with a flow rate of 0.65 mL/min with a mobile phase consisting of solution A (0.1% acetic acid in water) and B (acetonitrile) using the following linear gradient (time [min]; % B): (0;5), (0.25;5) (0.5;15), (1.0;20), (1.25;25) (1.9;35), (2.30;50), (2.50;50), (2.6;5). The total run time was 3.8 min. After each injection the sampling needle was washed with a washing solvent consisting of MeOH:H_2O (80:20 v/v). ESI Jetstream parameters were set as follows: drying gas temperature 275 °C, drying gas flow 5 L/min, nebulizer 45 psi, sheath gas temperature 320 °C, sheath gas flow 11 L/min, capillary entrance voltage 4000 V and nozzle voltage 1000 V. Quadrupoles Q1 and Q3 were set at the widest resolution (2.5 amu) and multiple reaction monitoring (MRM) mass transitions used for the quantification of recorded traces are presented in Supplementary Table S4.

Labeling of cathepsin X with DCG-04

PC-3 cell lysate was diluted in cathepsin X assay buffer (1 mg/ml protein concentration). Inhibitor was added (10 μM) and after 10 minutes at room temperature biotinylated probe DCG-04 was added (10 μM). Reaction was stopped at several time points by boiling the sample in SDS-loading buffer for 5 minutes[1]. Proteins were separated on 12% Tris-glycine gel and transferred to a Hybond-N nitrocellulose membrane using iBlot 2 dry blotting system (Thermo Scientific). After blocking with 5% skimmed milk powder in TBS with 0.05% Tween 20, membrane was incubated with streptavidin-HRP (1:5000; Jackson ImmunoResearch) for 1 hour and proteins were detected with

GeneSnap software (Syngene) using SuperSignal West Dura Extended Duration Substrate chemiluminescence kit (Thermo Scientific).

Supplementary Figures

Supplementary Figure S1. Relative inhibition of cathepsin X.

a) 579 compounds (first group) (50 μM) were screened for inhibition of cathepsin X. Values of five compounds with the highest relative inhibition are shown. An irreversible inhibitor of cysteine proteases, E-64, (10 μM) was used as a positive control. b) Second group of 20 compounds, with structures similar to those of inhibitors **1** and **2** were screened for cathepsin X inhibition. Values are the average of two independent experiments, performed in duplicate ± S.E.M.

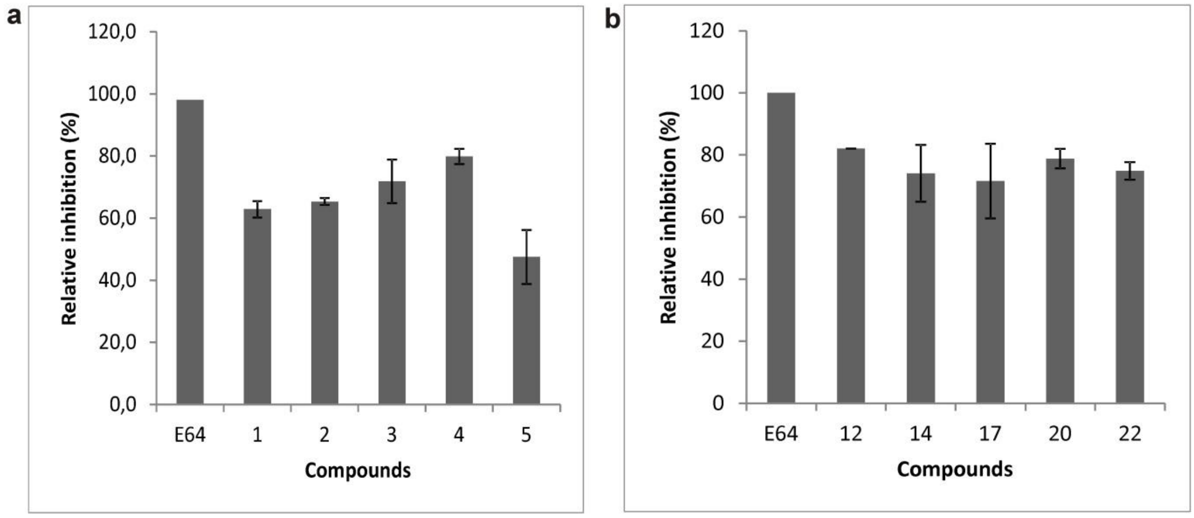

Supplementary Figure S2. Cytotoxicity of the inhibitors and their activities in cell lysates. PC-3 (a) and PC-12 (c) cells were treated with 10 μM inhibitors for 24 and 48 hours. Their cytotoxic effect was evaluated with MTS assay. The control group (DMSO) was considered as 100% of cell viability. Values are the average of three independent experiments, each performed in triplicate ± S.E.M. b) and d) Cells were treated with inhibitors (10 μM) for 24 hours. Cathepsin X activity was measured in PC-3 (b) and PC-12 (d) cell lysates (dark columns). Additionally, in another experiment lysates of non-treated cells were pre-incubated with inhibitors (10 μM) for 30 minutes and then cathepsin X activity measured (light columns). Values are the average of three independent experiments, performed in duplicate ± S.E.M. *P < 0.017 (T test).

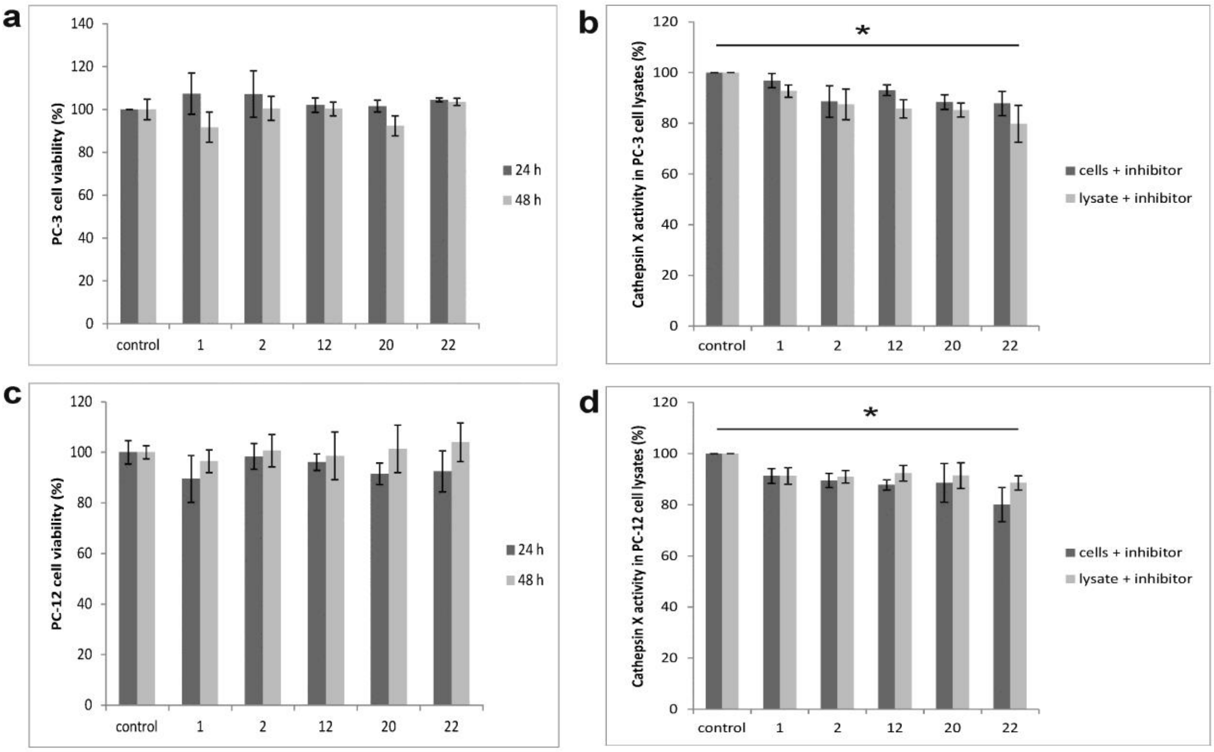

Supplementary Figure S3. Alternative setting of migration assay using cell invasion and migration (CIM) plates which excludes measurement of cell adhesion shows that compound 22 inhibits migration of PC-3 cells.

Since cathepsin X participates also in cell adhesion another setting of migration assay was performed using CIM plates. No inhibitor was added to the complete media of the lower compartments whereas the inhibitor was added to the serum-free media of the upper compartments after cells had attached (3 hours after seeding). The level of PC-3 cell migration was assessed by the increases in the curve slopes (1/h) that represent cell migration ability. Slopes were calculated from cell index values in the chosen time interval. Results are normalized to cells treated with DMSO (the control) with 100 % migration ability. Assays were carried out twice, each in quadruplicate. Results are means ± S.E.M. *P < 0.05 (T test).

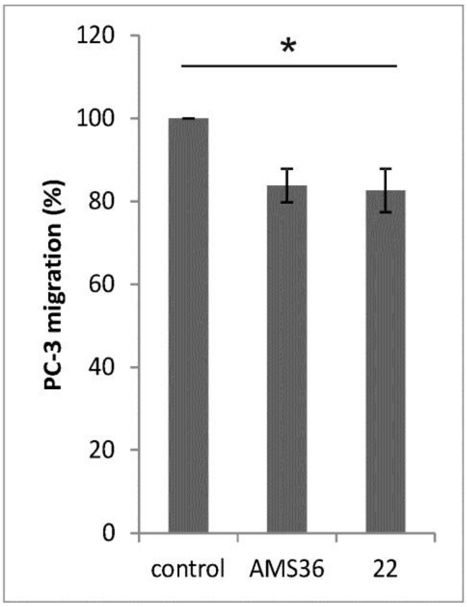

Supplementary Figure S4. Molecular docking of compound 22 in cathepsin X.

To estimate the possible binding conformations of compound **22** in the active site of cathepsin X AutoDock Vina software was used. For this purpose, the crystal structure of procathepsin X (PDB code: 1DEU)[2] with removed prodomain was used to study the pattern of interaction between **22** and the active site of the enzyme. Compound **22** fits into the V-shaped active site with the 2,3-dihydrobenzo[*b*][1,4]dioxine part inserted into the S2 pocket of cathepsin X. The score for the best conformation was –7.2 kcal/mol. The model suggests possible intermolecular hydrogen bonds between the carbonyl *O*-atom of **22** and the NH group of GLY-73, the triazol *N*2-atom of **22** and the OH group of TYR-27, the triazol *N*1- and *N*2-atom and the NH_2 group of GLN-22, the triazol *N*1-atom of **22** and the imidazol *Nε*-atom of HIS-23 and the triazol *N*1-atom of **22** and the indol *N*-atom of TRP-202. The same ligand-enzyme set was used for docking with FRED (OpenEye Scientific software) which returned basically the same conformation of compound **22** in the active site of cathepsin X (data not shown).

Red dashed lines represent the distance between *O*- and *N*-atoms of donors and acceptors, not the actual hydrogen bond length. On the surface representation of cathepsin X active site catalytic CYS-31 is colored in yellow.

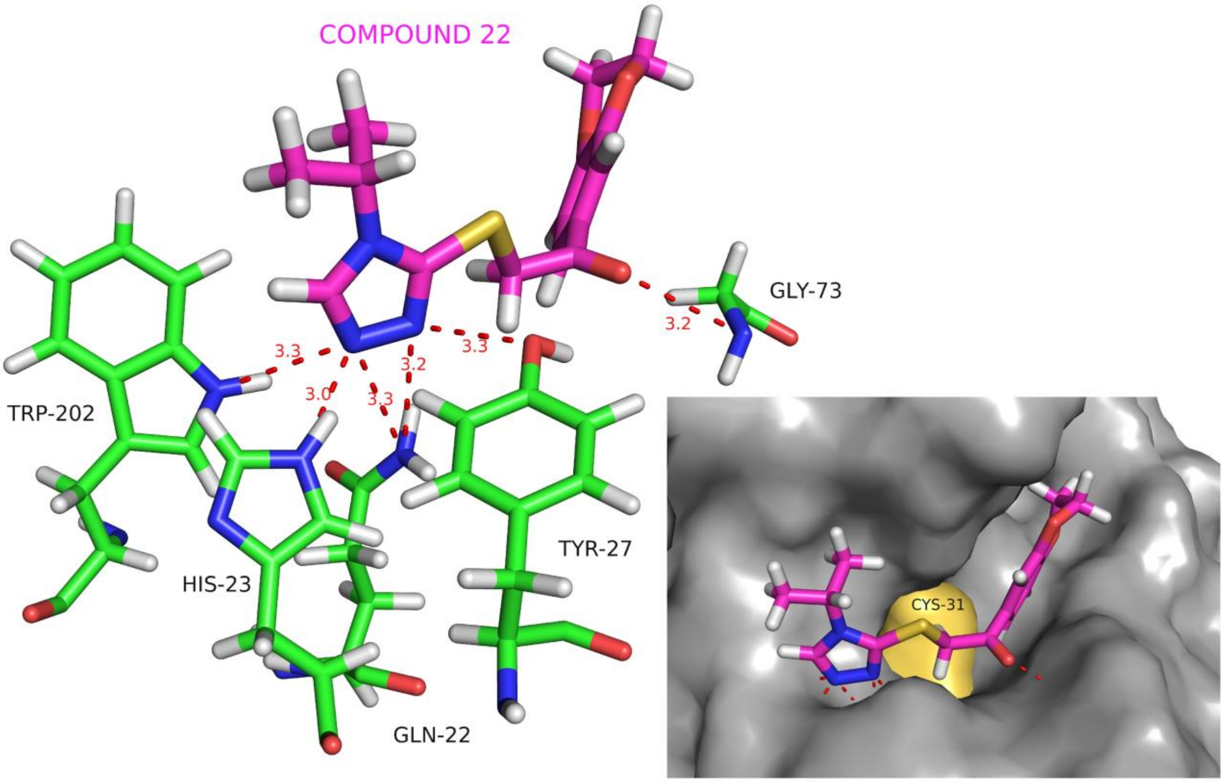

Supplementary Figure S5. Delayed cathepsin X labeling in the presence of reversible inhibitor.

Cell lysate was pre-treated with AMS36 (a) or compound **22** (b) and then labeled with biotinylated activity-based probe DCG-04. DMSO was used as a control. The reaction was stopped at different time points and labeling determined with SDS-PAGE and streptavidin-HRP western blot. a) Cathepsin X was strongly inhibited by irreversible inhibitor AMS36 through-out the experiment. AMS36 also inhibited cathepsin B. b) Cathepsin X labeling was delayed for approximately 20 minutes with compound **22** as is typical for reversible inhibitors. A weak inhibition of cathepsin B was also observed, but to a lesser extent as with AMS36. A representative image of two performed experiments is shown. Due to the chemiluminescent detection, size marker (SeeBlue® Pre-stained Protein Standard, Life Technologies) is added as a separate strip.

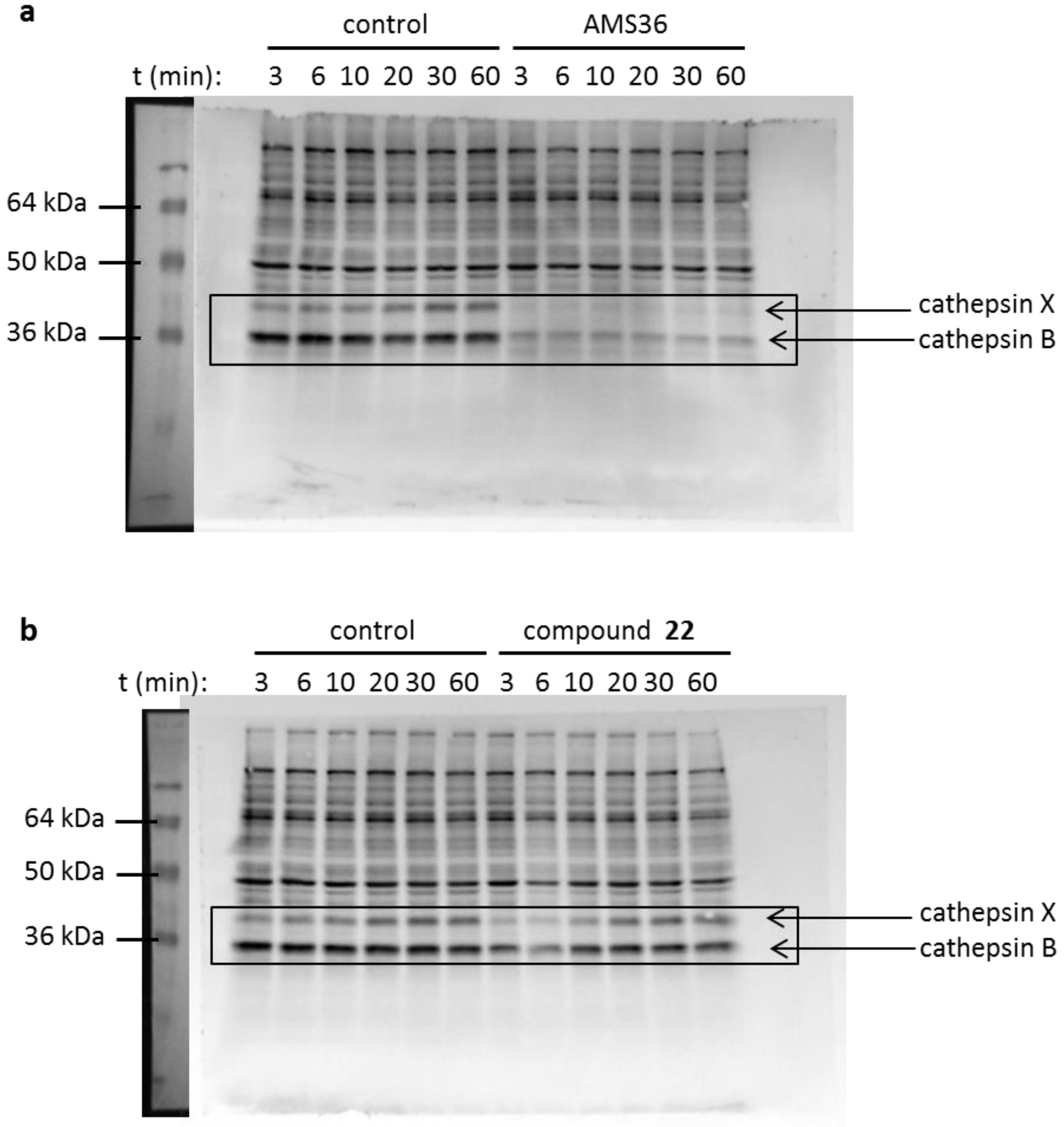

Supplementary Table S1. Compounds from the second group: structure and relative inhibition.

Compound	Structure	% inhibition (1 h preincubation)
**6**	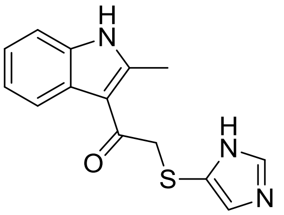	44 %
**7**	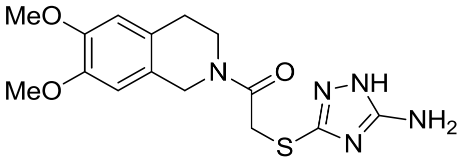	0 %
**8**	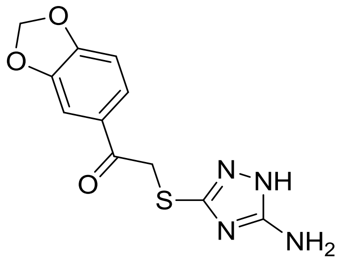	67 %
**9**	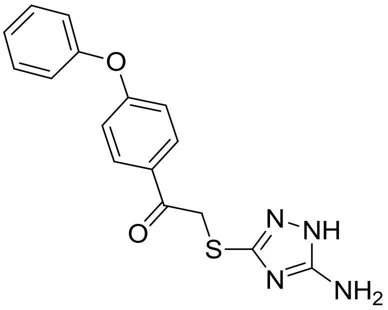	27 %
**10**	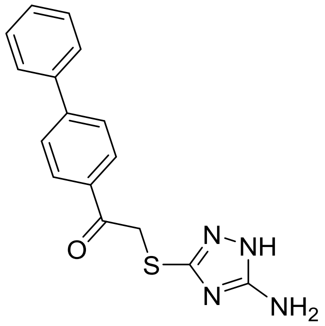	64 %

**11**	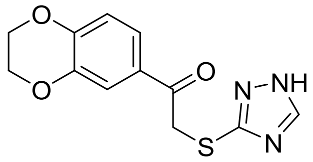	72 %
**12**	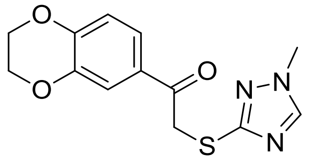	82 %
**13**	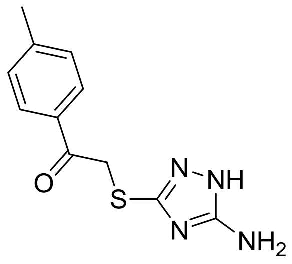	22 %
**14**	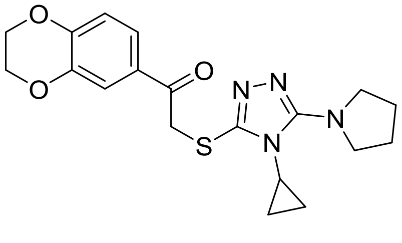	74 %
**15**	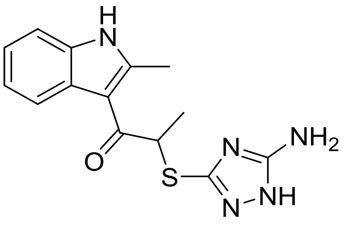	23 %
**16**	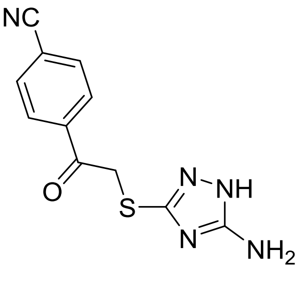	15 %
**17**	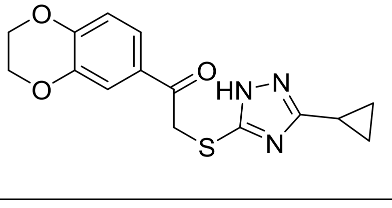	72 %

**18**	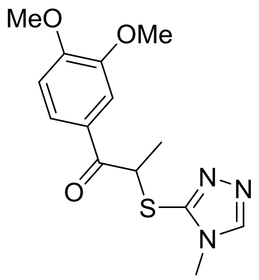	25 %
**19**	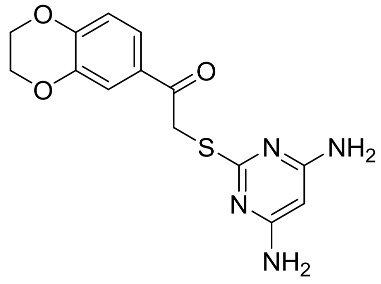	27 %
**20**	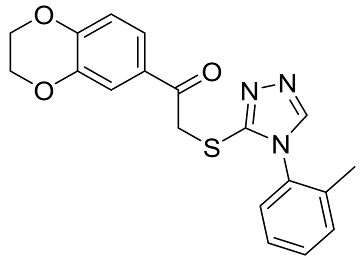	79 %
**21**	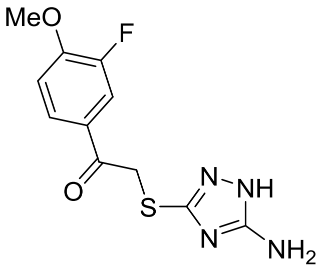	39 %
**22**	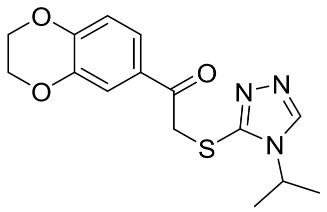	75 %

**23**	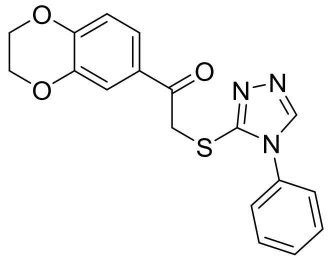	72 %
**24**	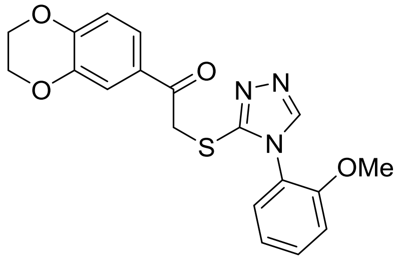	73 %
**25**	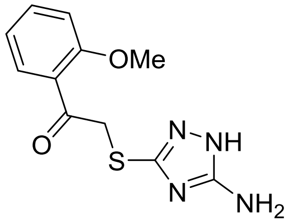	50 %

Supplementary Table S2. Substrates used for the various cathepsins and their concentrations for K_i determination.

Enzyme	Substrate	Concentration (μM)	Reference/Company
**cathepsin X**	Abz-FEK(Dnp)-OH	3.25, 6.5, 13	Puzer et al.[3]
**cathepsin B - endopept.**	Z-RR-AMC	60, 180, 360	Bachem
**cathepsin B - exopept.**	Abz-GIVRAK(Dnp)-OH	1, 3, 6	Bachem
**cathepsin S**	Z-VVR-AMC	0.5, 1, 1.5	Biomol
**cathepsin L**	Z-FR-AMC	0.5, 1, 4	Bachem
**cathepsin H**	Z-R-AMC	20, 80, 160	Biomol

Supplementary Table S3. Theoretical partition coefficient of selected triazole-based cathepsin X inhibitors.

Theoretical partition coefficient was calculated using ChemDraw software (PerkinElmer) and expressed as logP which represents theoretical prediction of the hydrophilic/lipophilic properties of the compounds. From the obtained values for membrane non-permeable inhibitor E64, its membrane permeable analogue E64d and our compounds, we can predict good membrane permeability for the selected inhibitors.

**Compound**	**logP**
E64	- 1.35
E64d	- 0.75
1	1.49
2	1.11
12	1.29
20	3.32
22	1.83

Supplementary Table S4. Cellular uptake and retention of compound 22.

Multiple reaction monitoring quantification settings for recorded analyte; m/z transitions printed in bold represent quantifier ions, while the normal text is used for qualifier ions.

**Analyte**	**Retention time (min)**	**MRM (*m/z*)**	**CE (eV)**	**Frag (V)**	**Polarity**
**22**	**2.04**	**320.1 > 142**	**17**	**91**	**+**
22	2.04	320.1 > 114	29	91	+

MRM, multiple reaction monitoring; CE, collision energy; Frag, fragmentor voltage;

Cellular uptake and residual amount of compound **22** in the cell media after 1 hour are shown.

**Inhibitor**	**Concentration in cells**	**% of 22 in the cell media**
22	7.1 µmoles/10^6 cells	84 %

References

1 Leung, D., Hardouin, C., Boger, D. L. & Cravatt, B. F. Discovering potent and selective reversible inhibitors of enzymes in complex proteomes. *Nat Biotechnol* **21**, 687-691 (2003).

2 Sivaraman, J., Nägler, D. K., Zhang, R., Ménard, R. & Cygler, M. Crystal structure of human procathepsin X: a cysteine protease with the proregion covalently linked to the active site cysteine. *J Mol Biol* **295**, 939-51 (2000).

3 Puzer, L. *et al.* Recombinant human cathepsin X is a carboxymonopeptidase only: a comparison with cathepsins B and L. *Biol Chem* **386**, 1191-1195 (2005).
